# Determinants of safety helmet use among motorcyclists in Kerala, India

**DOI:** 10.5249/jivr.v2i1.26

**Published:** 2010-01

**Authors:** Jayadevan Sreedharan, Jayakumary Muttappillymyalil, Binoo Divakaran, Jeesha C Haran

**Affiliations:** ^*a*^Research Division, Gulf Medical University, Ajman, UAE.; ^*b*^Department of Community Medicine, Academy of Medical Sciences, Kannur, Kerala, India – 670 503.; ^*c*^Department of Community Medicine, SMCSI Medical College, Karakkonam, Trivandrum, Kerala, India.

## Abstract

**Background:**

Motorcycles account for a large proportion of road traffic accidents in India and the riders of these vehicles run a high risk of injuries or death. This study aims to explore the determinants of helmet use among motorcyclists in Kerala, India.

**Methods:**

A cross-sectional study conducted in Kerala, India, over a period of six months. 309 motorcyclists in Kerala were interviewed for this study using a pretested structured questionnaire.

**Results:**

Among 309 motorcyclists, 80% were less than 40 years of age, and only 24% were females. Among the total, only 31.4% used a helmet. There was a statistically significant association between the use of helmet and gender, marital status, drunken driving, use of alcohol and attitude towards implementing legislative measures. Odds Ratios observed were 5.3 for female gender compared to male, 4.5 for those with a positive attitude towards the implementation of legislative measures on helmet use, 3.7 for those who were not drunk while driving and 2.3 for unmarried compared to married persons.

**Conclusions:**

The study concludes that the determinants associated with the practice of helmet use were gender, drunken driving, marital status and positive attitude towards legal measures.

## Introduction

With the rapid expansion of motor vehicle use in low- and middle-income countries, road traffic-related death and injuries are increasing sharply.^1^ Projections show that, between 2000 and 2020, road traffic deaths will decline by about 30% in high-income countries but increase substantially in low- and middle-income countries.^1^ Without appropriate action, by 2020, road traffic injuries are predicted to be the third leading contributor to the global burden of disease and injury.^1,2^ More than one million people died from road traffic crashes in low-and middle-income countries in 2000; according to the World Health Organization, that number could nearly double by 2020. Every day thousands of people are killed and injured in traffic accidents. Millions of people each year spend many weeks in hospital after severe crashes and many will never be able to make a living, work or play as in the past. More than half of the people killed in traffic crashes are young adults aged between 15 and 44 years and often individuals who support their family with their earnings. Motorcycle accidents account for a large proportion of road traffic accidents in India and the riders of these motorized vehicles have a high risk of injuries or death.^3,4^ Motorcycle accidents are associated with a high incidence of head injuries. Health care costs due to head injuries are not affordable for an average citizen in a country like India where health insurance is not that common.^5^ Evidence exists to suggest that use of helmets can reduce the risk and occurrence of both head injuries and death due to motorcycle accidents and in turn reduce hospitalization and morbidity.^6^ A study by Moskal et al observed that helmet use significantly decreased the risk of head and facial injuries. The study also reported that there was however no association between helmet use and the occurrence of neck or cervical spine injuries. It concluded that helmets protect users of motorized two-wheel vehicles against head and facial injury without increasing the risk of neck or cervical spine injury.^7^ A study among traffic accident victims in New Delhi by Banerjee et al. revealed that 31% were the victims of head injury and the study also suggests the need for wearing a helmet as a preventive strategy for head injury.^8^An important means of increasing the wearing of helmets in low- and middle-income countries is legislation; where helmet-wearing rates are low and a large number of people use motorized two-wheelers.^9^

There is considerable evidence to show that helmet use effectively reduces motorcycle-related head injuries. Helmet use lessens serious injuries, lowers mortality rates and reduces the need for hospital resources.^6^ The most specific and most effective way of reducing head injuries and fatalities resulting from motorcycle and bicycle crashes is the use of helmets. In most low- and middle-income countries, especially in Asia, a motorcycle is the common vehicle for the family.^9^ Helmet use among the motorcycle road users is low. Young motorcycle users in particular are generally less likely to wear a helmet than those who are older.^9^ A study in a Brazilian city, for instance, found that those younger than 18 years were less likely than others to wear a helmet, particularly if they had been consuming alcohol.^10^

But helmets are not uniformly worn by all motorcycle riders in Kerala due to various reasons. The purpose of the present study was to explore the determinants of helmet use among motorcyclists and to determine the prevalence of helmet use at the time of the study.

## Methods

This cross-sectional study was conducted in the Indian state of Kerala. Kerala State is situated in the south-west India. It is bounded by Karnataka State in the north, Tamilnadu in the south and east. It has a population of 31.8 million as per the 2001 census report. The state has a literacy rate of 97%, among the highest in India. According to the 2001 census of India figures, 56% of residents are Hindus, 24% Muslims, 19% Christians and the remaining 1% are of some other faith. This study was conducted over a period of six months from May to October 2007. A pre-tested structured closed-ended questionnaire was used for data collection. The questionnaire included socio-demographic characteristics like age, gender, education, marital status, religion and topics relating to their awareness of helmet use and attitudes towards helmet use and practice. 309 motorcyclists across Kerala were interviewed for this study. The sample size was calculated by considering the prevalence of helmet use as 30%, alpha = 0.05 and 20% error, thus the minimum sample size observed was 235. For the purpose of the study, the state was divided into three regions: south, central and north. From each region, a minimum of 100 samples were collected by random procedure. A random point in each region was identified and the motorcyclists riding past were asked to participate in the study. After obtaining informed consent from the motorcyclists, the questionnaire was administered. The purpose of the study was explained after providing the questionnaire to them. The data obtained were analysed using SPSS 17. Simple and multiple logistic regression with helmet use as the dependent variable and rider’s gender, religion, marital status, education, use of alcohol, use of mobile phone while driving, attitude towards the role of stringent legislative measures in the use of safety helmets were the independent variables. Chi-square test, simple and multiple logistic regression methods were performed. Tests were considered significant when the two-sided ‘p’ value was <0.05.

## Results

The present study involved a total of 309 randomly selected motorcyclists. There were 235 (76.1%) males and 74 (23.9%) females. Their ages ranged from 17 to 61 years with a mean age of 34.5 + 8.9 years. The maximum number of participants was in the age group 30-39 years. More than 70% of respondents were married and about 49% were educated up to graduate level or above, whereas only 6.5% had less than high school education. All respondents had some form of formal education. The details are given in Table 1

**Table:** Table 1: **Socio-demographic characteristics of respondents by gender**

Variable	Group	Male n=235 Number (%)	Female n=74 Number (%)	Total n=309 Number (%)	P value
Age Group (Years)	<30	53 (22.6)	22 (29.7)	75 (24.3)	<0.001
	30-39	124 (52.8)	48 (64.9)	172 (55.7)	
	>=40	58 (24.7)	4 (5.4)	62 (20.0)	
Marital status	Married	171 (72.8)	46 (62.2)	217 (70.2)	NS
	Unmarried	64 (27.2)	28 (37.8)	92 (29.8)	
Education	Below high school	20 (8.5)	--	20 (6.5)	<0.01
	High school or undergraduate	108 (46.0)	30 (40.5)	138 (44.7)	
	Graduate or above	107 (45.5)	44 (59.5)	151 (48.8)	
Total		235 (76.1)	74 (23.9)	309 (100)	

With regard to the prevalence of helmet use at the time of interview, only 83 (26.9%) of the motorcyclists were using a helmet. Of the 309 respondents, 130 (42.1%) said they were habitual alcohol users. It was observed that 184 (59.5%) respondents believed stringent legislative measures would promote the regular use of helmet by motorcyclists. In the present study, 36 (11.7%) respondents claimed ignorance about the need for a safety helmet. Ninety-seven (31.4%) riders however claimed they used a safety helmet while riding a motorcycle, but only 48 (49.5%) identified themselves as regular safety helmet wearers. The details are given in Table 2.

**Table:** Table 2: **Compliance with safety helmet use and opinion about stringent legislative measures by gender**

Variable	Group	Gender		Total
		Male	Female	
		Number (%)	Number (%)	
Opinion on regular safety helmet use	Yes	112 (47.7)	7 (9.5)	119
	No	123 (52.3)	67 (90.5)	190
Opinion on stringent legislative measures for use of safety helmet	Yes	138 (58.7)	46 (62.2)	184
	No	97 (41.3)	28 (37.8)	125

Among males, about 48% were using a helmet but among females only 9.5% were using a helmet at the time of interview. Fifty-nine percent of males and 62% of females were of the opinion that the introduction of stringent legislative measures for the implementation of helmet use will promote regular helmet use. From the model, the independent determinants observed were gender, positive attitude towards the implementation of legislative measures, drunken driving and marital status. Females displayed a 5.4 times higher chance of using helmet compared to males (p<0.001), Those with a positive attitude towards the implementation of legislative measures complied significantly more with helmet use than those with a negative attitude (p<0.001), those who had not drunk while driving were 3.7 times more likely to use a helmet and unmarried persons were 2.3 times more likely to use a helmet compared to married persons. The details are summarized in Table 3.

**Table:** Table 3: Independent factors associated with helmet use

Variable	Group	Regular safety helmet wearers		Total	p	OR#	CI€
		Yes (n=83)	No (n=226)				
		Number (%)	Number (%)				
Age	<40 years	68 (27.5)	179 (72.5)	247	0.60	--	--
	>=40 years	15 (24.2)	47 (75.8)	62		--	--
Gender	Male	48 (20.4)	187 (79.6)	235	<0.001	1	--
	Female	35 (47.3)	39 (52.7)	74		5.4	2.6-12
Religion	Hindu	43 (25.0)	129 (75.0)	172	0.38	--	--
	Christian	18 (34.6)	34 (65.4)	52		--	--
	Muslim	22 (25.9)	63 (74.1)	85		--	--
Marital status	Married	51 (23.5)	166 (76.5)	217	<0.05	1	--
	Unmarried	32 (34.8)	60 (65.2)	92		2.3	1.1–4.4
Educational attainment	Below high school	5 (25.0)	15 (75.0)	20	0.98	--	--
	High school or under-graduate	37 (26.8)	101 (73.2)	138		--	--
	Graduate or above	41 (27.2)	110 (72.8)	151		--	--
Use of alcohol	Yes	26 (20.0)	104 (80.0)	130	<0.05	1.3	0.5–3.2
	No	57 (31.8)	122 (62.8)	179		1	--
Use mobile phone while riding	Yes	20 (21.7)	72 (78.3)	92	0.20	--	--
	No	63 (29.0)	154 (71.0)	217		--	
Opinion on stringent legislative measures for use of safety helmet	Yes	66 (39.5)	118 (64.1)	184	<0.001	4.5	2.0–9.0
	No	17 (13.6)	108 (86.4)	125		1	--
*Drunken riding	Yes	8 (13.8)	50 (86.2)	58	<0.05	1	--
	No	52 (29.5)	124 (70.5)	176		3.7	1.2–11

* Only 234 participants responded to this question # Adjusted Odds Ratio € Confidence Interval

Respondents’ opinion of the major causes of motorcycle crashes include a combination of factors like a dangerous road, speeding, poor condition of the vehicle and a busy road or congestion.

## Discussion

Our study showed a prevalence of helmet use at the time of interview of 26.9%. The determinants of helmet use observed were gender, marital status, driving while drunk, use of alcohol and attitude towards implementing legislative measures in using helmet. According to the participants the major factors associated with motorcycle crashes were a combination of dangerous road, speeding, poor condition of the vehicle and busy road or congestion.

Various studies in different parts of the world have reported diverse safety helmet use rates. Hung et al. found 23% of Vietnamese motorcyclists were wearing a helmet at the time of interview in Vietnam.^11^ Sharma et al. observed that about 72.1% of motorcyclists reported 'not always' and 23.3% reported 'never' wearing a helmet in India.^12^ The study by Sharma et al. also observed that hazardous traffic behavior was found to be significantly greater among males and young riders.^12^ Another study from India reported that 69.8% of riders admitted never/very occasionally using a helmet.^3^ The findings from the present study also support the observations from many of the other studies.

Hung et al. conducted a study in Vietnam to investigate the barriers to and factors associated with helmet use. They observed safety helmet use among motorcyclists and reported that an array of factors were associated with helmet use like support for universal helmet legislation and a positive attitude towards the negative attributes of helmet use such as inconvenience and discomfort in hot weather.^11^ Other determinants observed were older age, being a driver, trips of greater than 10 km distance, higher levels of education.^11^ It also reported that over 95% of motorcyclists disagree with the statement that wearing a helmet does not reduce the severity of head injury in a crash, most motorcyclists believed that helmets need not be worn for a short trip.^11^

La Torre et al. conducted a study to estimate the incidence and related determinants of head injuries before and after the implementation of a new universal helmet law in Italy and concluded that helmet use has a protective effect on head trauma among scooter riders.^13^ O’Callaghan and Nausbaum investigated the determinants of helmet use among adolescents. The study concluded that by means of strengthening the routine use of helmets and building young people's confidence that they can overcome any perceived barriers to helmet use, helmet wearing behavior will increase.^14^ Dandona et al. in India observed that the significant predictors of helmet use included: driving a borrowed two wheeler; driving a moped/ scooterette/scooter as compared to a motorcycle; lower education; age >45 years; and male drivers.^3^ This study observed that the major determinants of helmet use were gender, marital status, positive attitude towards the implementation of legislative measures and not driving drunk. The findings therefore support the observations made from many of the studies conducted in different parts of the world.^11-14^

Legislation has been enacted in some countries to mandate helmet use by bicyclists and reports thereafter show that helmet use increased after the implementation of the law. A review done by Macpherson and Spinks concluded that bicycle helmet legislation appears to be effective in increasing helmet use and decreasing head injury rates.^15^The Vietnamese study also concluded that efforts to increase helmet use need to focus on the necessity for universal helmet legislation in association with identifying solutions to reduce the negative attitudes towards helmet use.^11^ La Torre et al reported that one year after implementing a universal law, helmet use has increased substantially and a sharp reduction in head injury among persons older than 18 years was seen.^13^ A study by Dandona et al. also suggests the need to enact and enforce policy interventions in order to improve the driving license system, mandatory use of a helmet, effective traffic law enforcement and ensuring good vehicle condition.^3^

## Conclusion

This study concluded that helmet use among motorcyclists was infrequent among the participants. Education and motivation on road safety measures are the two factors that have to be considered to improve helmet use among motorcyclists. The determinants associated with helmet use were gender, drunk driving, marital status and a positive attitude towards legal measures. The study highlights the importance of enforcing stringent legislative measures to improve helmet use by motorized two-wheeler riders. Periodic police checks are an essential component in enforcing helmet use. It requires a strong political will and concerted sustained efforts across a range of sectors. There is an urgent need for setting and enforcing laws requiring riders of motorized two wheelers to wear a helmet along with strict action to prevent people riding under the influence of alcohol.
